# Correlation between sedentary activity, physical activity and bone mineral density and fat in America: National Health and Nutrition Examination Survey, 2011–2018

**DOI:** 10.1038/s41598-023-35742-z

**Published:** 2023-06-21

**Authors:** Zhao Lin, Guang Shi, Xun Liao, Jingrou Huang, Mingyu Yu, Wei Liu, Xue Luo, Hongrui Zhan, Xiyu Cai

**Affiliations:** 1 Department of Orthopedics, The Fifth Affiliated Hospital of Sun Yat-sen University, Zhuhai, Guangdong Province China; 2 Department of Rehabilitation, The Fifth Affiliated Hospital of Sun Yat-sen University, Zhuhai, 519000 Guangdong Province China

**Keywords:** Health care, Medical research

## Abstract

We compared the relationship between sedentary activity (SA) and physical activity (PA) with bone mineral density (BMD) and body fat percentage in the United States and found a negative association between SA and BMD and a positive association with body fat percentage. A positive association between PA and BMD and a negative association with body fat percentage. SA and PA are associated with changes in skeletal parameters and body fat percentage, and we aimed to investigate and compare the relationship between SA, PA and bone mineral density (BMD) and body fat percentage in men and women. We assessed the relationship between SA, PA and BMD and body fat percentage in 9787 Americans aged 20–59 years (mean age 38.28 ± 11.39 years) from NHANES 2011–2018. BMD and body fat percentage were measured by dual-energy X-ray bone densitometry (DXA). We used multiple linear regression models to examine the relationships between SA, PA and lumbar spine BMD and total body fat percentage, adjusted for a large number of confounding factors. After adjusting for race/ethnicity, age, alcohol and smoking behavior, body mass index (BMI), total protein, blood calcium, blood uric acid, cholesterol, blood phosphorus, vitamin D, and blood urea nitrogen, SA was negatively associated with lumbar spine BMD (β = − 0.0011 95% CI − 0.0020 to − 0.0002, *P* = 0.022), and SA was positively associated with total fat percentage (β = PA was positively associated with lumbar BMD (β = 0.0046 95% CI 0.0010 to 0.0082, *P* = 0.012) and there was a negative association between PA and body fat percentage (β = − 1.177 95% CI − 1.326 to –1.027, *P* < 0.001). Our results show that physical activity is a key component of maintaining bone health in both men and women and is strongly associated with lower body fat percentages. Sedentary activity is negatively correlated with bone density and is strongly associated with an increase in body fat percentage. Healthcare policy makers should consider reducing sedentary activity and increasing physical activity when preventing osteoporosis and obesity.

## Introduction

Osteoporosis is characterized by the deterioration of the microstructure of bone tissue and reduced bone density, which increases the risk of skeletal fractures^1,2^. In the USA, osteoporosis cost $57 billion in 2018, which is projected to grow to over $95 billion per year by 2040^3^. Considering the global increases in life expectancy and the burden of osteoporosis fractures on societies, health systems, and individuals, effective osteoporosis prevention strategies are essential.

Bone mineral density (BMD) decreases following peak bone mass due to multifaceted and complex changes in sex hormones, nutrition, and bone loading^4^. Modifiable behaviors, such as smoking^5^, dietary intake^6^, and exercise^7^, can contribute to osteoporosis development in old age. As a result of inactivity and reduced weight-bearing loads, such as bed rest^8^ and time in reduced gravity^9^, bone turnover and mineral homeostasis are altered. In previous studies, physical activity (PA) and sedentary activity (SA) were associated with different effects on BMD in females and males^10^. Physical activity is recommended for the management of osteoporosis by the guidelines^11^. It is controversial, however, whether such interventions have any effect on people who do not have osteoporosis, i.e., those who are seeking prevention of osteoporosis. It is crucial to provide a summary of the evidence in this field so that specific recommendations can be made regarding PA/SA engagement for osteoporosis prevention^12^.

In 2019, Kim et al.^10^ used data from the Korea National Health and Nutrition Examination Survey (NHANES) and found that PA correlates positively with hip BMD in men. There was no association between PA and BMD at any site in females. A systematic review has shown that physical activity is very protective against the reduction of bone mineral density in the lumbar spine^12^. Interestingly, recent studies have found an association between low BMD and SA (such as sitting in front of a TV or the internet) among adolescents^13,14^. In addition, according to NHANES 2005–2006, there was a negative correlation between repeated exposure to SA and femoral and hip BMD, independent of the number of times women engaged in moderate and vigorous activity^15^. In a meta-analysis, four studies reported a significant positive association between SA and BMD, and two reported a significant negative association. Five studies reported no correlation between SA and BMD in males^16^. Thus, a potential association between objectively measured SA/PA and BMD in adulthood needs to be further investigated.

People who are overweight or obese tend to have an increased risk of various life-threatening diseases (including cardiovascular disease (CVD), diabetes and even cancer) and increased mortality^17^. Several studies have shown that high body fat percentage is an independent risk factor for CVD, coronary events^18^ and all-cause mortality^19,20^. Some evidence suggests an association between PA and SA and body fat percentage, but previous studies have reported inconsistent results across age groups.

In conclusion, SA/PA is associated with BMD and body fat percentage, but the evidence for their association is ambiguous. A nationally representative cohort should be used to determine the relationship between SA/PA and BMD and percent body fat in men and women and will provide key information for prevention and treatment strategies for osteoporosis and obesity. Therefore, we used data from NHANES to assess the relationship between SA/PA and BMD and body fat percentage in Americans.

## Materials and methods

### Study design and population

**Figure 1:**
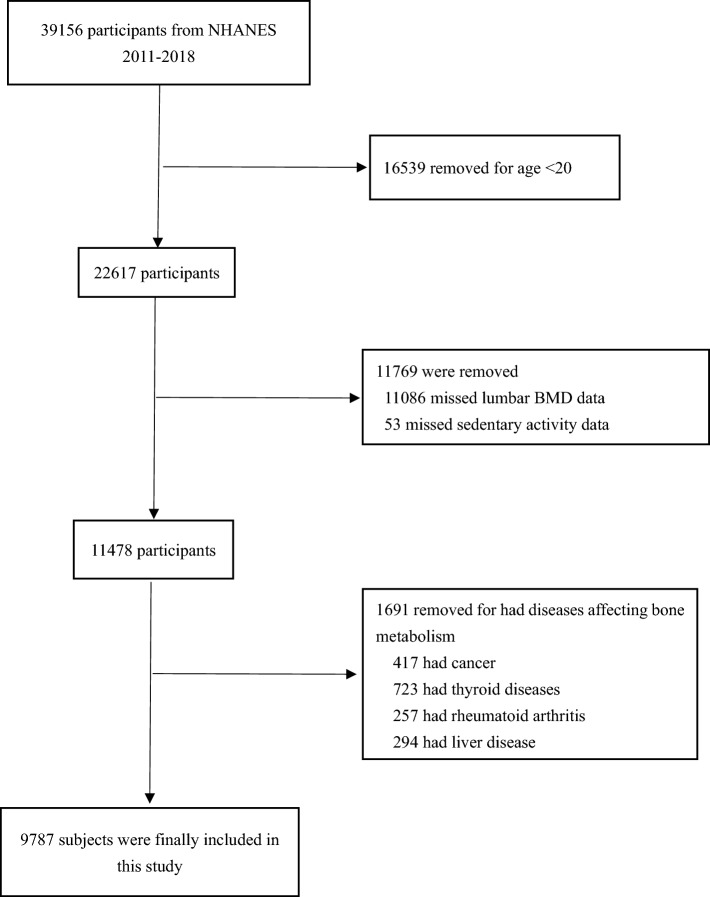
Flowchart of participants’ selection.

In this study, the data we analyzed were drawn from the National Health and Nutrition Examination Survey (NHANES), a nationally representative survey of the U.S. population conducted through a complex, multistage, probability sampling design that provides information on the general health and nutritional status of the civilian, noninstitutional population of the United States. The design, data collection procedures, sample weight and informed consent have been described in detail at the National Center for Health Statistics, from which related data can be publicly available. Our analysis combined data from the NHANES cycles 2011–2012, 2013–2014, 2015–2016 and 2017–2018. A total of 39,156 subjects were initially included, of which those aged < 20 years (n = 16,539), those with missing information on lumbar spine BMD measurements (n = 11,086), and those with missing information on sedentary activity time (n = 53) were excluded from the study. There were also subjects with diseases affecting BMD (n = 1691) (including cancer patients (n = 417), thyroid disease (n = 723), rheumatoid arthritis patients (n = 257) and liver disease (n = 294) were excluded from the study. Ultimately, 9,787 eligible subjects were included in the study. The participant selection flow chart is shown in Fig. 1.

### Variables

The main variables in this study were SA (independent variable), PA (independent variable), and lumbar spine BMD (dependent variable) and total fat percentage (dependent variable). SA and PA were collected at home by trained interviewers using a structured questionnaire from the Computer Assisted Personal Interviewing (CAPI) system. The PA questionnaire was based on the Global Physical Activity Questionnaire (GPAQ) and provided respondent-level physical activity level data. Sedentary activity was measured by counting the number of hours per day that subjects were sedentary. Physical activity was measured by counting the sum of time spent in vigorous recreational activity and moderate recreational activity in a month for each subject and averaging this into daily activity time. BMD and fat percentage were measured by dual energy x-ray absorptiometry, measured by DXA (Hologic QDR 4500A fan-beam densitometer). Covariates were selected based on previous studies reporting risk factors for BMD, including sociodemographic variables, and blood biochemical characteristics. Questionnaire information was used to obtain information on sex, age, race/ethnicity (non-Hispanic white, non-Hispanic black, Mexican American, Other Hispanic, other race), PIR, physical activity (sedentary, physical activity time), education (Less than 9th grade; 9th–11th grade. High school graduate, college degree or above), alcoholic (no or), and smoker (no or). Comorbidities including thyroid disease, rheumatoid arthritis, liver disease, and malignancy were obtained by self-reported physician diagnosis. Key variables involving body measurements of weight, height and body mass index (BMI) were calculated by dividing weight (kg) by height squared (m^2^). Blood biochemicals include total protein, blood calcium, cholesterol, blood phosphorus, blood urea nitrogen, vitamin D, and SUA. For more information about the SA, PA, BMD, and fat measurement process and the process of obtaining other covariates, please visit http://www.cdc.gov/nchs/nhanes/.

### Statistical analyses

All data were derived from the National Health Service Board sample weights, as the goal of the NHSA is to generate data representative of the civilian noninstitutionalized population in the United States. We performed statistical analyses according to CDC guidelines (https://wwwn.cdc.gov/nchs/nhanes/tutorials/default.aspx). We first processed missing data for covariates: for categorical variables (education, physical activity, drinking status, and smoking status), missing data were considered as a separate group. For missing continuous variables, the corresponding means were used to complement In addition, given the complexity of the survey design, sample weights were considered in the statistical analysis according to CDC guidelines. Characteristics of the study population were expressed as weighted means (standard error, Se) and weighted percentages of continuous variables. Multiple regression analysis was applied to assess the independent correlations between SA, PA and BMD and fat percent. Smooth curve fitting was used to account for the nonlinear relationships between SA, PA and BMD and fat percent. Subgroup analysis was performed using a weighted generalized additive model. All calculations were performed with the R package (http://www.R-project.org, The R Foundation) and (http://www.empowerstats.com, X&Y Solutions, Inc, Boston, MA), and we used percentages of categorical variables and means ± standard deviations of continuous variables. *P* values less than 0.05 (two-sided) were considered statistically significant.

## Results

Our study included 9787 Americans aged 20–59 years with a mean age of 38.28 ± 11.39. In this study, 46.26% were female, 14.84% were Mexican American, 9.96% were other Hispanic, 32.86% were non-Hispanic white, 23.23% were non-Hispanic black, and 19.11% were other races (including multi race). The duration of SA was divided into four groups (Q1: < 4 h; Q2: 4–5.5 h; Q3: 5.5–7 h; Q4: ≥ 7 h), as shown in Table  1. The baseline characteristics of the four groups were significantly different except for total serum calcium, serum phosphorus and cholesterol. Participants in the highest SA group were more often male, non-Hispanic white, had higher education, had higher PIR and BMI, lower total serum protein, and higher serum uric acid.

**Figure 2:**
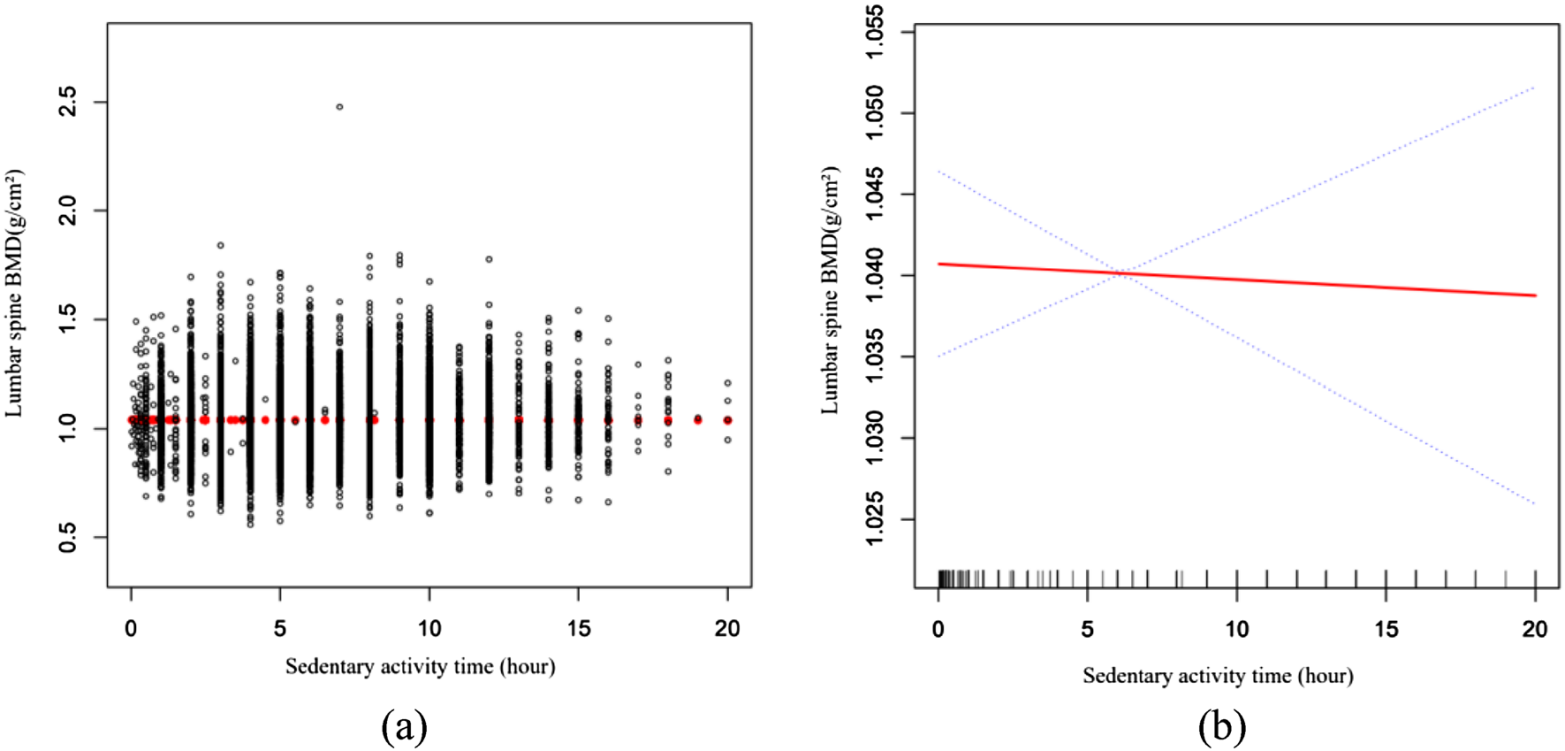
The association between sedentary activity time and lumbar Spine BMD. (**a**) Each black point represents a sample. (**b**) Red line represents the smooth curve fit between variables. Blue lines represent the 95% of confidence interval from the fit. age, race/Hispanic origin, gender, drinking behavior, smoking behavior, BMI, PIR, total protein, serum calcium, cholesterol, serum phosphorus, blood urea nitrogen, Vitamin D, physical activity and SUA were adjusted.

The results of the multivariate regression analysis are detailed in Table 2. After adjusting for confounders, there was a negative correlation between SA and lumbar BMD (β = − 0.0011, 95% CI − 0.0020 to – 0.0002, *P* = 0.022). Converting SA from a continuous variable to a categorical variable (four subgroups), individuals in the highest SA group had 0.0091 g/cm^2^ lower BMD than individuals in the lowest group (β = − 0.0091, 95% CI − 0.0173 to − 0.0009, *P* = 0.028). Figure 2 shows a smoothed curve fit of the relationship between SA and total BMD.

**Table 1:** Characteristics of the study population based on sedentary activity time quartiles.

sedentary activity time	Total	Q1(< 4 h)	Q2(4–5.5 h)	Q3(5.5-7 h)	Q4(> 7 h)	*P* value
Number of subjects (n)	9787	2437	2327	1459	3564	
Age (years)	38.28 ± 11.39	39.10 ± 10.94	38.04 ± 11.65	37.43 ± 11.66	38.23 ± 11.37	< 0.001
BMI (kg/m^2^)	28.84 ± 6.89	28.50 ± 6.42	28.70 ± 6.76	29.10 ± 7.31	29.05 ± 7.09	0.166
PIR	2.51 ± 1.58	2.10 ± 1.42	2.36 ± 1.49	2.51 ± 1.60	2.89 ± 1.64	< 0.001
Lumbar Spine BMD (g/cm^2^)	1.04 ± 0.15	1.03 ± 0.15	1.04 ± 0.16	1.04 ± 0.16	1.04 ± 0.15	0.036
Blood urea nitrogen (mmol/L)	4.50 ± 1.54	4.57 ± 1.49	4.47 ± 1.54	4.47 ± 1.43	4.49 ± 1.61	0.010
Serum total calcium (mmol/L)	2.34 ± 0.08	2.33 ± 0.08	2.34 ± 0.08	2.35 ± 0.08	2.35 ± 0.08	< 0.001
Cholesterol (mmol/L)	4.93 ± 1.01	4.94 ± 1.03	4.92 ± 0.99	4.92 ± 1.07	4.93 ± 0.98	0.650
Serum phosphorus (mmol/L)	1.20 ± 0.18	1.19 ± 0.18	1.19 ± 0.18	1.20 ± 0.18	1.21 ± 0.18	< 0.001
Total protein (g/L)	72.00 ± 4.29	72.02 ± 4.33	72.18 ± 4.42	72.07 ± 4.37	71.85 ± 4.15	0.041
Serum uric acid (umol/L)	319.33 ± 80.26	312.54 ± 78.19	316.45 ± 79.77	323.11 ± 81.12	324.30 ± 81.22	< 0.001
Vitamin D (umol/L)	59.31 ± 24.03	60.15 ± 23.34	59.34 ± 23.02	59.10 ± 24.76	58.81 ± 24.83	0.022
Physical activity time (h)	0.76 ± 0.90	1.09 ± 1.18	0.90 ± 0.96	0.68 ± 0.76	0.49 ± 0.52	< 0.001
Gender(%)						0.497
Men	5260 (53.74%)	1322 (54.25%)	1264 (54.32%)	795 (54.49%)	1879 (52.72%)	
Women	4527 (46.26%)	1115 (45.75%)	1063 (45.68%)	664 (45.51%)	1685 (47.28%)	
Race/Hispanic origin (%)						< 0.001
Mexican American	1452 (14.84%)	551 (22.61%)	385 (16.54%)	182 (12.47%)	334 (9.37%)	
Other Hispanic	975 (9.96%)	351 (14.40%)	248 (10.66%)	110 (7.54%)	266 (7.46%)	
Non-Hispanic White	3216 (32.86%)	682 (27.99%)	732 (31.46%)	494 (33.86%)	1308 (36.70%)	
Non-Hispanic Black	2274 (23.23%)	494 (20.27%)	539 (23.16%)	362 (24.81%)	879 (24.66%)	
Other Race—Including Multi-Racial	1870 (19.11%)	359 (14.73%)	423 (18.18%)	311 (21.32%)	777 (21.80%)	
Education level (%)						< 0.001
Less than 9th grade	615 (6.28%)	295 (12.11%)	168 (7.22%)	58 (3.98%)	94 (2.64%)	
9–11th grade	1176 (12.02%)	413 (16.95%)	319 (13.71%)	151 (10.35%)	293 (8.22%)	
High school graduate	2156 (22.03%)	628 (25.77%)	572 (24.58%)	321 (22.00%)	635 (17.82%)	
College degree or above	5838 (59.65%)	1101 (45.18%)	1266 (54.40%)	929 (63.67%)	2542 (71.32%)	
Not reported	2 (0.02%)	0 (0.00%)	2 (0.09%)	0 (0.00%)	0 (0.00%)	
4/5 or more drinks every day (%)						< 0.001
Yes	2682 (27.40%)	798 (32.75%)	675 (29.01%)	383 (26.25%)	826 (23.18%)	
No	5478 (55.97%)	1174 (48.17%)	1298 (55.78%)	837 (57.37%)	2169 (60.86%)	
Not reported	1627 (16.62%)	465 (19.08%)	354 (15.21%)	239 (16.38%)	569 (15.97%)	
Smoked at least 100 cigarettes in life (%)						0.004
Yes	3694 (37.74%)	971 (39.84%)	909 (39.06%)	561 (38.45%)	1253 (35.16%)	
No	6089 (62.22%)	1464 (60.07%)	1418 (60.94%)	897 (61.48%)	2310 (64.81%)	
Not reported	4 (0.04%)	2 (0.08%)	0 (0.00%)	1 (0.07%)	1 (0.03%)	

Mean ± SD for continuous variables: the *P* value was calculated by the weighted linear regression model. (%) for categorical variables: the* P* value was calculated by the weighted chi-square test.

*SUA* serum uric acid, *PIR* poverty income ratio, *BMD* bone mineral density, *BMI* body mass index.

**Table 2:** The association between sedentary activity time(hour) and lumbar BMD (g/cm^2^).

	Model 1 β (95% CI) *P* value	Model 2 β (95% CI) *P* value	Model 3 β (95% CI) *P *value
Sedentary activity time(hour)	− 0.0001 (− 0.0010, 0.0008) 0.827	− 0.0009 (− 0.0018, − 0.0000) 0.041	− 0.0011 (− 0.0020, − 0.0002) 0.022
Sedentary activity time categories			
Q1(< 4 h)	Reference	Reference	Reference
Q2(4–5.5 h)	− 0.0015 (− 0.0103, 0.0072) 0.735	− 0.0065 (− 0.0150, 0.0021) 0.139	− 0.0066 (− 0.0152, 0.0019) 0.128
Q3(5.5–7 h)	− 0.0026 (− 0.0124, 0.0073) 0.610	− 0.0102 (− 0.0199, − 0.0006) 0.038	− 0.0109 (− 0.0206, − 0.0011) 0.029
Q4(> 7 h)	− 0.0003 (− 0.0081, 0.0075) 0.934	− 0.0075 (− 0.0153, 0.0002) 0.056	− 0.0091 (− 0.0173, − 0.0009) 0.029
* P* for trend	0.990	0.077	0.036
Subgroup analysis stratified by gender			
Men	− 0.0007 (− 0.0019, 0.0005) 0.260	− 0.0012 (− 0.0024, 0.0000) 0.055	− 0.0011 (− 0.0024, 0.0002) 0.087
Women	0.0007 (− 0.0006, 0.0019) 0.292	− 0.0004 (− 0.0016, 0.0008) 0.498	− 0.0009 (− 0.0022, 0.0003) 0.155
Subgroup analysis stratified by age			
20–34	− 0.0018 (− 0.0031, − 0.0005) 0.006	− 0.0022 (− 0.0035, − 0.0010) < 0.001	− 0.0015 (− 0.0029, − 0.0002) 0.026
35–49	0.0018 (0.0003, 0.0032) 0.015	0.0008 (− 0.0007, 0.0022) 0.296	0.0008 (− 0.0007, 0.0023) 0.293
≥ 50	− 0.0002 (− 0.0022, 0.0019) 0.882	− 0.0016 (− 0.0036, 0.0005) 0.142	− 0.0036 (− 0.0058, − 0.0015) 0.001
Subgroup analysis stratified by race/ethnicity			
Mexican American	0.0010 (− 0.0011, 0.0031) 0.338	0.0006 (− 0.0015, 0.0027) 0.582	0.0002 (− 0.0020, 0.0024) 0.859
Other Hispanic	0.0015 (− 0.0013, 0.0043) 0.304	0.0011 (− 0.0017, 0.0039) 0.441	− 0.0000 (− 0.0030, 0.0029) 0.977
Non-Hispanic white	− 0.0017 (− 0.0032, − 0.0002) 0.025	− 0.0016 (− 0.0031, − 0.0001) 0.032	− 0.0019 (− 0.0035, − 0.0003) 0.018
Non-Hispanic black	− 0.0003 (− 0.0023, 0.0017) 0.800	− 0.0001 (− 0.0021, 0.0019) 0.902	0.0003 (− 0.0018, 0.0023) 0.811
Other race—including multi-racial	− 0.0001 (− 0.0021, 0.0019) 0.921	− 0.0006 (− 0.0026, 0.0014) 0.548	0.0001 (− 0.0019, 0.0022) 0.887

Model 1: no covariates were adjusted. Model 2: age and race/ethnicity were adjusted. Model 3: age, race/Hispanic origin, gender, drinking behavior, smoking behavior, BMI, PIR, total protein, serum calcium, cholesterol, serum phosphorus, blood urea nitrogen, Vitamin D, physical activity, SUA. Abbreviation: SUA: serum uric acid. PIR: poverty income ratio. BMI: body mass index.

We then stratified by sex, age, and race/ethnicity, and after stratified analysis, only a negative association was found in non-Hispanic whites (β = − 0.0019 95% CI − 0.0035 to − 0. 0003, *P* = 0.018), the 20–34 years age group (β = − 0.0015 95% CI − 0.0029 to − 0.0002, *P* = 0.026), and ≥ 50 years (β = − 0.0036 95% CI − 0.0058 to − 0.0015, *P* = 0.001) the negative association between sedentary activity time and lumbar spine BMD remained significant. Gender was not a correcting factor for this relationship. Subsequently, we also performed a multiple linear regression analysis to explore the relationship between physical activity time and lumbar spine BMD (Table 3). We found a strong positive association between physical activity time and lumbar spine BMD. Stratified by sex, age, and race/ethnicity, we found a positive association between physical activity time and lumbar spine BMD in men (β = 0.0066 95% CI 0.0020 to 0.0111, *P* = 0.004) and Other Race—Including Multi-Racial (β = 0.0160 95% CI 0.0067 to 0.0253, *P* < 0.001) remained significant, and age was not a correcting factor for this relationship.

We also conducted multiple regression analysis on the relationship between SA time, PA time and bone mineral density in multiple parts of the body, and the results are shown in Table 4. We found that SA time was negatively correlated with BMD in multiple parts of the body, while PA time was positively correlated with BMD in multiple parts of the body. Then, we performed multiple linear regression analysis to explore the relationship between SA time, PA time and body fat percentage in multiple parts of the body (Table 5). We found a positive correlation between SA time and body fat percentage at multiple sites, and a strong negative correlation between PA and body fat percentage at multiple sites.

**Table 3:** The association between physical activity time (hour) and lumbar Spine BMD (g/cm^2^).

	Model 1 β (95% CI) *P* value	Model 2 β (95% CI) *P* value	Model 3 β (95% CI) *P* value
Physical activity time (hour)	0.0048 (0.0014, 0.0082) 0.006	0.0032 (− 0.0001, 0.0066) 0.057	0.0046 (0.0010, 0.0082) 0.012
Physical activity time (hour) categories			
Q1(≤ 0.136 h)	Reference	Reference	Reference
Q2(> 0.136, ≤ 0.375 h)	0.0216 (0.0123, 0.0308) < 0.001	0.0209 (0.0118, 0.0300) < 0.001	0.0197 (0.0107, 0.0288) < 0.001
Q3(> 0.375, < 1 h)	0.0158 (0.0063, 0.0253) 0.001	0.0132 (0.0039, 0.0226) 0.005	0.0133 (0.0038, 0.0227) 0.006
Q4(> = 1 h)	0.0183 (0.0089, 0.0278) < 0.001	0.0138 (0.0045, 0.0231) 0.004	0.0156 (0.0056, 0.0255) 0.002
*P* for trend	0.002	0.040	0.014
Subgroup analysis stratified by gender			
Men	0.0075 (0.0033, 0.0118) < 0.001	0.0068 (0.0027, 0.0110) 0.001	0.0066 (0.0020, 0.0111) 0.004
Women	0.0025 (− 0.0036, 0.0087) 0.422	− 0.0011 (− 0.0072, 0.0049) 0.717	0.0003 (− 0.0060, 0.0066) 0.924
Subgroup analysis stratified by age			
20–34	0.0069 (0.0024, 0.0113) 0.002	0.0063 (0.0020, 0.0106) 0.004	0.0043 (− 0.0004, 0.0091) 0.073
35–49	− 0.0015 (− 0.0074, 0.0044) 0.620	− 0.0020 (− 0.0077, 0.0038) 0.507	0.0060 (− 0.0001, 0.0122) 0.055
≥ 50	0.0043 (− 0.0054, 0.0141) 0.383	0.0055 (− 0.0042, 0.0152) 0.263	0.0061 (− 0.0041, 0.0162) 0.242
Subgroup analysis stratified by race/ethnicity			
Mexican American	0.0015 (− 0.0049, 0.0078) 0.652	0.0017 (− 0.0047, 0.0082) 0.598	0.0019 (− 0.0048, 0.0085) 0.582
Other Hispanic	− 0.0006 (− 0.0105, 0.0093) 0.902	− 0.0005 (− 0.0108, 0.0097) 0.917	0.0007 (− 0.0098, 0.0112) 0.897
Non-Hispanic white	0.0042 (− 0.0017, 0.0101) 0.162	0.0044 (− 0.0016, 0.0104) 0.147	0.0046 (− 0.0019, 0.0111) 0.167
Non-Hispanic black	0.0067 (− 0.0009, 0.0144) 0.085	0.0031 (− 0.0046, 0.0109) 0.426	0.0045 (− 0.0036, 0.0126) 0.280
Other race—including multi-racial	0.0172 (0.0087, 0.0256) < 0.001	0.0178 (0.0092, 0.0263) < 0.001	0.0160 (0.0067, 0.0253) < 0.001

Model 1: no covariates were adjusted. Model 2: age and race/ethnicity were adjusted. Model 3: age, race/Hispanic origin, gender, drinking behavior, smoking behavior, BMI, PIR, total protein, serum calcium, cholesterol, serum phosphorus, blood urea nitrogen, Vitamin D, sedentary activity, SUA

*SUA* serum uric acid, *PIR* poverty income ratio, *BMI* body mass index.

**Table 4:** The association between SA time(hour), PA time (hour) and bone mineral density (g/cm^2^).

	Model 1 β (95% CI) *P* value	Model 2 β (95% CI) *P* value	Model 3 β (95% CI) *P* value
SA time (h)			
Total BMD	− 0.0007 (− 0.0014, − 0.0001) 0.023	− 0.0012 (− 0.0018, − 0.0005) < 0.001	− 0.0015 (− 0.0021, − 0.0009) < 0.001
Lumbar spine BMD	− 0.0001 (− 0.0010, 0.0008) 0.827	− 0.0009 (− 0.0018, − 0.0000) 0.041	− 0.0011 (− 0.0020, − 0.0002) 0.022
Thoracic spine BMD	− 0.0006 (− 0.0012, 0.0001) 0.075	− 0.0006 (− 0.0013, 0.0000) 0.062	− 0.0013 (− 0.0020, − 0.0007) < 0.001
Trunk bone BMD	− 0.0010 (− 0.0017, − 0.0004) 0.001	− 0.0013 (− 0.0020, − 0.0007) < 0.001	− 0.0020 (− 0.0026, − 0.0014) < 0.001
Pelvis BMD	− 0.0009 (− 0.0019, 0.0000) 0.060	− 0.0011 (− 0.0020, − 0.0001) 0.025	− 0.0020 (− 0.0030, − 0.0011) < 0.001
Left arm BMD	− 0.0010 (− 0.0016, − 0.0005) < 0.001	− 0.0014 (− 0.0020, − 0.0008) < 0.001	− 0.0015 (− 0.0019, − 0.0011) < 0.001
Left leg BMD	0.0004 (− 0.0004, 0.0012) 0.320	− 0.0002 (− 0.0010, 0.0006) 0.626	− 0.0014 (− 0.0021, − 0.0006) < 0.001
PA time (h)			
Total BMD	0.0132 (0.0108, 0.0157) < 0.001	0.0124 (0.0099, 0.0148) < 0.001	0.0054 (0.0029, 0.0078) < 0.001
Lumbar spine BMD	0.0048 (0.0014, 0.0082) 0.006	0.0032 (− 0.0001, 0.0066) 0.057	0.0046 (0.0010, 0.0082) 0.012
Thoracic spine BMD	0.0076 (0.0051, 0.0101) < 0.001	0.0080 (0.0055, 0.0105) < 0.001	0.0022 (− 0.0003, 0.0048) 0.081
Trunk bone BMD	0.0127 (0.0102, 0.0151) < 0.001	0.0106 (0.0082, 0.0130) < 0.001	0.0041 (0.0016, 0.0066) 0.001
Pelvis BMD	0.0137 (0.0100, 0.0174) < 0.001	0.0110 (0.0074, 0.0147) < 0.001	0.0043 (0.0005, 0.0081) 0.026
Left arm BMD	0.0205 (0.0183, 0.0226) < 0.001	0.0204 (0.0182, 0.0226) < 0.001	0.0052 (0.0035, 0.0068) < 0.001
Left leg BMD	0.0202 (0.0170, 0.0233) < 0.001	0.0193 (0.0162, 0.0224) < 0.001	0.0061 (0.0033, 0.0090) < 0.001

Model 1: no covariates were adjusted. Model 2: age and race/ethnicity were adjusted. Model 3: age, race/ethnicity, gender, drinking behavior, smoking behavior, body mass index, PIR, total protein, serum calcium, serum uric acid, cholesterol, serum phosphorus, blood urea nitrogen, Vitamin D and SUA were adjusted.

*PIR* poverty income ratio, *BMI* body mass index.

**Table 5:** The association between SA time(hour),PA time (hour) and fat.

	Model 1 β (95% CI) *P* value	Model 2 β (95% CI) *P* value	Model 3 β (95% CI) *P* value
SA time (h)			
Left arm percent fat	0.2544 (0.1879, 0.3209) < 0.001	0.2828 (0.2157, 0.3498) < 0.001	0.1383 (0.1074, 0.1693) < 0.001
Left leg percent fat	0.1894 (0.1321, 0.2466) < 0.001	0.2026 (0.1444, 0.2607) < 0.001	0.1036 (0.0714, 0.1358) < 0.001
Right arm percent FAT	0.2346 (0.1699, 0.2993) < 0.001	0.2649 (0.1997, 0.3301) < 0.001	0.1303 (0.0994, 0.1611) < 0.001
Right leg percent fat	0.1787 (0.1219, 0.2355) < 0.001	0.1901 (0.1324, 0.2478) < 0.001	0.1070 (0.0747, 0.1392) < 0.001
Total percent fat	0.1882 (0.1380, 0.2384) < 0.001	0.2223 (0.1719, 0.2726) < 0.001	0.0942 (0.0693, 0.1191) < 0.001
Total fat	0.3481 (0.2797, 0.4165) < 0.001	0.3743 (0.3058, 0.4428) < 0.001	0.1131 (0.0884, 0.1379) < 0.001
PA time (h)			
Left arm percent fat	− 2.451 (− 2.700, − 2.202) < 0.001	− 2.392 (− 2.643, − 2.142) < 0.001	− 1.701 (− 1.903, − 1.499) < 0.001
Left leg percent fat	− 2.028 (− 2.245, − 1.811) < 0.001	− 2.058 (− 2.277, − 1.839) < 0.001	− 1.443 (− 1.633, − 1.254) < 0.001
Right arm percent FAT	− 2.353 (− 2.596, − 2.110) < 0.001	− 2.301 (− 2.545, − 2.056) < 0.001	− 1.585 (− 1.781, − 1.388) < 0.001
Right leg percent fat	− 1.971 (− 2.187, − 1.754) < 0.001	− 1.992 (− 2.210, − 1.773) < 0.001	− 1.368 (− 1.558, − 1.179) < 0.001
Total percent fat	− 1.757 (− 1.949, − 1.565) < 0.001	− 1.716 (− 1.908, − 1.525) < 0.001	− 1.177 (− 1.326, − 1.027) < 0.001
Total fat	− 1.211 (− 1.474, − 0.948) < 0.001	− 1.197 (− 1.459, − 0.935) < 0.001	− 0.720 (− 0.830, − 0.609) < 0.001

Model 1: no covariates were adjusted. Model 2: age and race/ethnicity were adjusted. Model 3: age, race/ethnicity, gender, drinking behavior, smoking behavior, body mass index, PIR, total protein, serum calcium, serum uric acid, cholesterol, serum phosphorus, blood urea nitrogen, Vitamin D and SUA were adjusted.

*PIR* poverty income ratio, *BMI* body mass index.

## Discussion

Our cross-sectional study investigated whether SA and PA were independently associated with lumbar BMD and adiposity in the US population using a large, nationally representative sample from the NHANES database. We found a negative association between sedentary activity and lumbar spine BMD and a positive association between sedentary activity and adiposity. Physical activity was positively associated with BMD, and physical activity was negatively associated with adiposity.

Osteoporosis is a multifactorial disease associated with nutritional, exercise, medical and genetic factors, and osteoporosis and osteoporotic fractures produce a heavy burden of disability and economic costs^21,22^. Previous studies have shown a negative association between SA and BMD; consistent evidence has been reported in older adults^23,24^. A prospective study from Brazil found that increased sitting time was associated with decreased lumbar BMD in women^25^. A study from the UK of men in northeastern England also found a negative association between sedentary time and spinal BMD in men^24^. However, another study from the University of British Columbia showed no observed independent effects of SA time on bone structure, bone density or strength in men and women (*P* > 0.05)^26^. Another study using NHANES (2005–2006) data did not find an association between SA and lumbar spine BMD in men or women, which is inconsistent with our findings, and the inconsistency may be due to differences in sample size, differences in SA time collection methods, and differences in study design and statistical methods^15^. Many previous studies have demonstrated the relationship between physical activity and lumbar spine BMD^10,24,27^. The results of one study on adolescents support the importance of moderate to severe PA as a positive factor in the accumulation of bone mass in adolescents^10^. Another study using NHANES (2005–2006) found that while there was no significant correlation between moderate to severe PA and BMD in young adults, in older adults, those with a longer duration of PA had higher BMD^28^.

With this large cross-sectional study, we demonstrated a negative association between SA and BMD and a positive association between various physical activities and BMD. Increased SA is often accompanied by increased indoor activity, resulting in reduced sunlight exposure and disruption of skeletal homeostasis^29^. Studies by Kim et al. have also found that sedentary behavior leads to hormonal responses, including the overproduction of the parathyroid hormone, that disrupt calcium metabolism required for bone formation^30,31^. The human skeleton is always in a bone formation-reabsorption equilibrium, and mechanical loading from exercise or weight bearing promotes bone health, while excessive sedentary activity disrupts this equilibrium and thus negatively affects bone health^15^. Sedentary activity may also have a negative impact on periosteal attachment, which is weakened by a decrease in continuously associated mechanical stimulation, which can also lead to bone loss. Increased SA time may also represent a decrease in PA time, which has been identified as an important stimulus for osteogenesis in previous studies^32^, and PA produces dynamic mechanical loads that affect bone through ground reaction forces and muscle contraction activity affecting the skeleton^33^. Wolfe's law describing bone formation under mechanical loading emphasizes the concept of a coupled association of muscle on bone remodeling^32^, with possible gender differences due to higher muscle mass in men.

Our study also found that the effect of physical activity on BMD was more pronounced in men. The results of the univariate analysis (*P* for trend, *P* < 0.05) also support a dose–response relationship between PA and BMD, i.e. those who are more active have higher BMD. Although it has been suggested that high levels of intense physical activity may be accompanied by a physiological process that overwhelms the osteogenic stimulatory effects of physical activity, the strenuous recreational activity reported by NHANES refers to high aerobic intensity activity in the general population, not high impact intensity activity in athletes^34,35^. Despite these speculations and findings, the exact mechanism of the correlation between SA, PA and BMD cannot be determined and requires further study.

With this large cross-sectional study, we demonstrated a positive association between SA and percentage body fat, and a negative association between physical activity and percentage body fat. Obesity contributes to increased mortality and a higher risk of cardiovascular disease, diabetes and cancer^17^. Body mass index (BMI) is often used as a strong indicator of normal weight, overweight and obesity, but healthy individuals with high muscle mass may also be misclassified as overweight or even obese^36^. Body fat percentage and physical activity correlate more consistently than BMI. Our study found a strong negative correlation between PA and percent body fat and a strong positive correlation between SA and percent body fat. In addition, an additional hour of physical activity per day was inversely associated with trunk adiposity and this effect was more pronounced than total adiposity^37^. This is consistent with the conclusion we obtained, suggesting that the relationship between physical activity and body fat percentage varies across body parts. Maher and colleagues investigated the relationship between physical activity and sedentary time and obesity based on BMI using NHANES data from 2003 to 2006, and similar to our results, the authors concluded that physical activity was strongly associated with BMI, while sedentary time had no significant results^38^.This may be due to the fact that physical activity not only reduces fat mass but also increases muscle mass, while sedentary activity is more associated with fat accumulation.

The strength of this study is that it uses a large sample analysis of the NHANES survey, and the data are highly reliable and standardized to be representative of the general U.S. population. In addition, we stratified the analysis according to sex, age and race to make the results more detailed and reliable. There is no denying the following limitations of our study. First, this is a cross-sectional study, and therefore, causality cannot be inferred. Further longitudinal studies with strong evidence are needed to address the causality of these relationships. Second, we did not analyze the relationship between SA, PA, and femoral BMD due to limited data, as insufficient data would have led to incomplete results. Finally, we may not have adjusted for variables that may bias the results, such as calcium intake and dietary intake.

## Conclusions

Our findings suggest that there is a negative association between sedentary activity and BMD and a positive association with body fat percentage in the US population. In contrast, there was a positive association between physical activity and BMD and a negative association with body fat percentage. Confounding factors such as race may influence these associations. More research is needed on the relationship between SA, PA and BMD and body fat percentage, including specific mechanisms and confounding factors associated with adjustment. In the meantime, clinical guideline developers should consider the positive effects of recommended physical activity on BMD and the beneficial associations of lowering body fat percentage when developing osteoporosis and obesity prevention strategies.
